# Systematic Analysis of Differential H3K27me3 and H3K4me3 Deposition in Callus and Seedling Reveals the Epigenetic Regulatory Mechanisms Involved in Callus Formation in Rice

**DOI:** 10.3389/fgene.2020.00766

**Published:** 2020-07-17

**Authors:** Nannan Zhao, Kang Zhang, Chunchao Wang, Hengyu Yan, Yue Liu, Wenying Xu, Zhen Su

**Affiliations:** ^1^State Key Laboratory of Plant Physiology and Biochemistry, College of Biological Sciences, China Agricultural University, Beijing, China; ^2^Hebei Key Laboratory of Plant Physiology and Molecular Pathology, College of Life Sciences, Hebei Agricultural University, Baoding, China

**Keywords:** callus, ChIP-seq analysis, H3K27me3, H3K4me3, rice, seedling

## Abstract

Plant growth and development occurs through meristematic cell activity, and cell fate transition is accompanied by epigenetic modifications. Callus with cell pluripotency exhibits the ability to undergo continued cell division, and is ideal for studying plant meristematic differentiation. By comparing the differential epigenetic modifications between callus and seedling, the changes in chromatin state and effects of various epigenetic modifications on the growth and development of plants can be revealed, and the key genes related to plant growth and development can be identified, providing novel insights into the regulation of plant growth and development. In this study, we performed ChIP assays using various antibodies in rice seed-induced callus and seedlings grown for about 15 days to examine the differential deposition of H3K27me3 and H3K4me3. Furthermore, data for DNase I-hypersensitive sites in the corresponding tissues were downloaded from National Center for Biotechnology Information. We analyzed 4,562 callus H3K27me3-decreased genes especially those encoding transcription factors in callus, and found that most of the transcription factors, including AP2-ERREBP, NAC, and HB gene families, were related to growth and development. Genes related to meristemization, such as *OsWOX9*, *OsWOX11*, *OsPLT4*, *OsPLT5*, and *OsSHR*, were also included. In contrast, H3K4me3 positively regulated callus characteristics through its higher deposition in the callus than in the seedling. We further performed transcriptomic analysis on 45 sets of Affymetrix GeneChip arrays and identified 1,565 genes preferentially expressed in the callus. Callus development and root development in rice were found to share a common regulatory mechanism. We found that these genes, which are associated with meristems, require the removal of H3K27me3 and the deposition of H3K4me3, and DNase I-hypersensitive sites to maintain a relatively active state in the callus than in the seedling. The present study provides novel data about the epigenetic mechanisms involved in callus formation and additional resources for the study of cell division and differentiation in plants.

## Introduction

Plants possess the remarkable ability to undergo cellular dedifferentiation and regeneration (Lee and Seo, 2018). Generally, plant tissue is cultured on specific media, which enables cells to retain the ability to develop into intact individuals. The genome in the callus, a highly proliferative cell mass that has long been inferred to be dedifferentiated, undergoes reprogramming to restore stem cell status and acquire pluripotency during callus formation, and the callus is therefore capable of direct organ formation and embryogenesis (Ikeuchi et al., 2013). The callus in rice is usually seed-induced, a group of transparent, dense cell clusters that retain the ability to undergo division and differentiation. Embryogenic callus mainly contains a set of homogeneous pluripotent cells, and represents an *in vitro* model of meristematic tissue. The cell division, differentiation, and embryogenic properties of callus are consistent with those of the corresponding biological processes *in vivo*.

Callus formation is accompanied by a change in cell fate during the process of the development of a whole plant: in this process, the epigenetic patterns of the cell also change accordingly, thereby altering chromatin structure and regulating gene expression (Lee and Seo, 2018). Generally, dedifferentiated cells exhibit a relatively open chromatin state corresponding to epigenetic modifications associated with gene activation. Conversely, differentiated cells generally have a relatively tight chromatin state that corresponds to inhibition of gene expression (Chen and Dent, 2014; Lee et al., 2016). Epigenetic modifications, e.g., methylation and acetylation, can define the expression of specific genes in multiple tissues to ensure tissue-specific gene expression (Chen and Dent, 2014).

Histone lysine methylation plays an important regulatory role in gene expression and in altering chromatin structure. Trimethylation of histone H3 Lys-27 (H3K27me3) plays a critical role in regulating gene expression during plant and animal development (Makarevitch et al., 2013). H3K27me3 is an epigenetic modification that inhibits gene expression; previous studies have shown that H3K27me3 regulates the expression of genes involved in the wound response, root stem cells, and development of embryonic characteristics (Xu et al., 2012; Liu et al., 2014; Kareem et al., 2015). In *Arabidopsis*, H3K27me3 undergoes dynamic changes during leaf-to-callus transition, that is, in callus, some auxin pathway-related genes and root development regulation-related genes show a decreasing trend of H3K27me3; however, an increasing trend for the accumulation of this epigenetic modification is observed near genes related to leaf development (He et al., 2012). H3K27me3 shows strong tissue specificity in the regulation of gene expression and plays a crucial role in regulating plant growth and development. In rice, WOX11 interacts with H3K27me3 demethylase JMJ705 to activate gene expression during shoot development. WOX11 and JMJ705 jointly control shoot growth and regulate the expression of a group of genes involved in chloroplast biosynthesis, and energy metabolism in meristematic tissues (Cheng et al., 2018). H3K27me3 is present in multiple tissues with tissue-specific effects, and many genes acquire or lose this methylation mark during cell differentiation, demonstrating the dynamic regulation of this epigenetic modification in response to plant development and environmental signals.

H3K4me3, in contrast to H3K27me3, acts primarily to activate gene expression. H3K4me3 has been shown to play an important role in the regulation of gene expression during plant growth and development. Disruption of the level of H3K4me3 affects the stability of the entire genome (Avramova, 2009; Berr et al., 2011). In rice, the H3K27 methyltransferase gene *SDG711* and the H3K4 demethylase gene *JMJ703* have antagonistic functions in reprogramming the H3K27me3/H3K4me3 ratio and regulating gene expression in the inflorescence meristem (Liu et al., 2015).

Bivalent chromatin comprises segments of DNA bound to both repressing and activating epigenetic marks in the same regions. The existence of bivalent modifications was first found in pluripotent mouse embryonic stem cells (Azuara et al., 2006; Bernstein et al., 2006). Generally, bivalent domains are defined by the coexistence of a permissive histone mark (H3K4me3) and a repressive mark (H3K27me3). Bivalent domains tend to coincide with transcription factor (TF) -encoding genes expressed at low levels (Bernstein et al., 2006). In animals, the pluripotency is dependent on the maintenance of appropriate epigenetic landscapes; generally, bivalent domains are preferentially present in undifferentiated embryonic stem cells (Mikkelsen et al., 2007). In *Arabidopsis*, the H3K4me3 and H3K27me3 marks are present in the genes *FLC* and *AP1*, suggesting that bivalent chromatin may be a characteristic of plant developmental regulatory genes (Saleh et al., 2008).

Open chromatin structure, which usually contains promoters, enhancers and insulators, facilitates and allows the binding of TFs. These regions, have been shown to be marked by DNase I-hypersensitive sites (DHSs) and lack nucleosomes or exhibit modification and replacement of dynamic nucleosomes. In rice, epigenetic maps of DHSs from callus and seedling have been described, and the differentially expressed genes (DEGs) in the seedling and callus were shown to be frequently associated with DHSs in both tissues (Zhang et al., 2012). As a histone variant, H2A.Z in the callus and seedling was also mapped, and Gene Ontology (GO) analysis showed that genes regulated by H2A.Z may be involved in environmental responses, chromatin assembly, and cell cycle in the callus (Zhang et al., 2017b).

Epigenetic modifications play various roles in the specific biological processes that occur during callus formation. In *Arabidopsis*, the AP2/ERF transcription factors, i.e., the WIND genes that are involved in triggering callus formation and subsequent plant regeneration (Iwase et al., 2011), are under the control of Polycomb Repressive Complex 2 (PRC2)-dependent repressive histone modification (Ikeuchi et al., 2015). In *Arabidopsis*, callus formation is a necessary step in the regeneration of new plants from isolated plant tissues, and the callus characteristics resemble those of root meristems (He et al., 2012). The (AUXIN RESPONSE FACTOR) ARF-LATERAL ORGAN BOUNDARIES DOMAIN (LBD) module is involved in root development (Okushima et al., 2007), whereas during callus formation, the ARF and LBD genes are also rapidly induced through ATXR2-mediated H3K36me3 deposition. In addition, the histone acetyltransferases GENERAL CONTROL NON-DEREPRESSIBLE 5 (GCN5) and SET DOMAIN GROUP 2 (SDG2), which are necessary for genome–wide H3K4me3 deposition, regulate the expression of *PLT1* and *PLT2* to establish the root stem cell niche (Kornet and Scheres, 2009; Yao et al., 2013). The cell cycle is also required for active callus formation, and CDK inhibitors are regulated by PROPORZ1 (PRZ1)-mediated H3ac accumulation (Cheng et al., 2015). Another group of genes, which includes *LEC1* and *LEC2*, regulates the reacquisition of embryonic characteristics; these genes are repressed via a complex epigenetic regulatory network. The LEC genes are repressed by PRC-mediated H3K27me3 and H2AK119ub deposition (Bratzel et al., 2010; Bouyer et al., 2011). In addition, the histone deacetylases HDA6 and HDA19 are recruited to the LEC genes in the differentiated tissue to maintain a differentiated state (Zhou et al., 2013).

Although the activity of the callus is critical for plant transduction, it remains unclear how chromatin and epigenetic modifications regulate callus activity in rice. In this study, we conducted whole-genome H3K27me3 and H3K4me3 ChIP-Seq experiments on the callus in the undifferentiated state and in seedlings that had already undergone differentiation, and downloaded data for DHSs of corresponding tissues from GEO database in National Center for Biotechnology Information (NCBI). The DHS data were used to study the roles of individual epigenetic modifications in the formation of callus and the regulatory mechanisms of key developmental genes. We additionally identified 1,565 genes that were preferentially expressed in the callus to reveal how multiple epigenetic modifications regulate callus characteristics. In addition, valuable genetic data sets that provide novel insights into various epigenetic regulatory mechanisms of plant regeneration were obtained.

## Materials and Methods

### Plant Materials and Growth Conditions

Seeds of rice (“Nipponbare”) were germinated in water for 3 days at 35°C, and then grown in a glasshouse for 19 days (12 h light and 12 h dark; 28/26°C). Rice callus tissue was induced from sterilized ‘Nipponbare’ seeds in rice callus induction medium at 28°C for 15 days. For the purposes of this study, rice seedling tissue refers to the shoot parts.

### Chromatin Immunoprecipitation

Chromatin immunoprecipitation (ChIP) was performed as described previously, with minor modifications (Nagaki et al., 2003). Approximately 15 g of tissue was homogenized to a fine powder in liquid nitrogen and re-suspended in TBS [10 mM Tris, pH 7.5, 3 mM CaCl_2_, 2 mM MgCl_2_, 0.1 mM PMSF, 2/5 tablet of complete mini (Roche Applied Science^1^) with 0.5% Tween 40]. The nuclei were purified in a sucrose gradient and digested with micrococcal nuclease (MNase; Sigma–Aldrich^2^). The nucleosome samples were first incubated with pre-immune rabbit serum (1: 100 dilution) and then with 4% protein A Sepharose (GE Healthcare life Sciences^3^) for 2 h, after which they were centrifuged at 13,000 rpm at 4°C using a low temperature centrifuge type MIKR200R from Hettich Lab Technology. The supernatant was incubated with anti-trimethyl-histone H3 (Lys 4) (H3K4me3; 07-473; Merck Millipore^4^) and anti-trimethyl-histone H3 (Lys 27) (H3K27me3; 07-449; Millipore) antibodies at 4°C overnight. An equal quantity of pre-immune rabbit serum, which served as a non-specific binding control in each ChIP experiment, was used in the control experiments. The samples were incubated with 25% protein A Sepharose at 4°C for 2 h. After centrifugation, the pellet (bound) fractions were subjected to a series of washes and the immune complexes were eluted from the washed beads using elution buffer. Immunoprecipitated DNA was extracted using phenol/chloroform and precipitated with ethanol. The ChIPed DNA was used for library construction and sequencing by the Beijing Genomics Institute.

### ChIP-Seq Data Analysis

Sequence reads were mapped to the reference genome of rice (MSU Rice Genome Annotation Release 6.1) using BOWTIE 2 (Langmead and Salzberg, 2012) under default parameters. The MACS program was used to shift the reads to identify peaks and convert the data to wiggle (WIG) format (bandwidth, 300 bp; model fold, 10, 30; *P* = 1.00e-5) (Zhang et al., 2008). WIG files were visualized with the UCSC genome browser (Kent et al., 2002). Reads were aligned with the reference genome and peak numbers are shown in Supplementary Table 1. The distribution of peaks identified in the ChIP-Seq and DNase-Seq data along the rice genome were characterized using CEAS software (Shin et al., 2009; Du et al., 2013). After the positions of the peaks were determined, genes (including the 2-kb upstream and gene body regions) overlapping the peaks were considered to carry the epigenetic marks (Zhang et al., 2017a).

### SOM Analysis

Self-organizing map (SOM) analysis is performed using two processes, training and mapping. First, the training process is completed using in-house and publicly obtained samples of diverse modification types that have been integrated into a plant chromatin state database (PCSD^5^) (Liu et al., 2018). Then, the mapping process is completed by inputting the wiggle files, which are processed by MACS 1.4.1 (Zhang et al., 2008) to analysis. The comparison between the two SOM maps was performed by the diffmap program in ERANGE software (Mortazavi et al., 2013; Yan et al., 2019).

### RNA-Seq Data and Analysis

The RNA was extracted using TRIZOL reagent (Invitrogen, now Thermo Fisher Scientific) and purified using Qiagen RNeasy columns (Qiagen^6^). The sequencing libraries were constructed by the Beijing Genomics Institute and sequenced using an Illumina HiSeq^TM^ 2,500, following standard protocols.

The reads were mapped to the rice reference genome of MSU version 6.1 using TOPHAT 2.0.10 (Trapnell et al., 2009) with the default parameters. The FPKM values (fragments per kilobase of transcript per million mapped reads) were calculated by CUFFLINKS 2.2.1 (Trapnell et al., 2010) with default parameters. Genes with an expression fold change >2 were filtered as differentially expressed.

### Gene Ontology Enrichment Analysis

Gene Ontology enrichment analysis was performed using the agriGO website (Du et al., 2010; Tian et al., 2017) and REVIGO (Supek et al., 2011). GO terms with a *P*-value less than 0.05 were considered significantly enriched.

### Statistical Analysis of Affymetrix GeneChip Data

Forty-five Affymetrix GeneChip arrays of 13 tissues and 17 developmental stages were used to analyze the callus preferentially expressed genes; data were normalized to the same level, and the *P*-value cut-off was selected as 0.05.

## Results

### Genome-Wide Differential Profiling of H3K27me3 Between Rice Callus and Seedling

**FIGURE 1:**
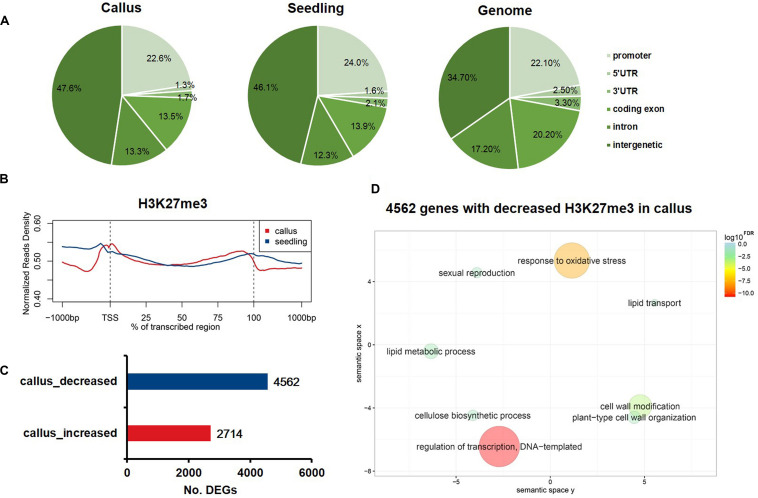
The characteristics of H3K27me3 in rice callus and seedling tissue. **(A)** The genomic distribution of H3K27me3 peaks within different regions in rice callus and seedling tissues. The rice genome was characterized into six classes that included five genic regions [promoter, 5′ untranslated region (UTR), 3′ UTR, coding exon, and intron] and intergenic regions. **(B)** The distribution of H3K27me3 along all the rice genes in the callus and seedling (from 1-kb upstream regions to 1-kb downstream regions). **(C)** The number of genes with differential deposition of H3K27me3 between callus and seedling. **(D)** GO enrichment analysis of 4,562 H3K27me3-decreased genes in callus by agriGO and REVIGO. The scatterplot shows the cluster representatives in a two-dimensional space derived by applying multidimensional scaling to a matrix of significant GO terms with semantic similarities. Bubble color and size indicate the log10^*FDR*^.

To profile the genome-wide distribution of H3K27me3 in rice, we conducted the ChIP-Seq (chromatin immunoprecipitation with massively parallel DNA sequencing) experiment in callus and seedling. We mapped the sequenced reads onto the whole genome and classified the whole genome into six regions, namely promoter, 5′UTR, 3′UTR, coding exon, intron, and intergenic regions, to observe the differential deposition of H3K27me3 among various genome elements (Figure 1A). The whole genome mapping rate was relatively high, up to 98.27% and 96.51% in the callus and seedling, respectively (Supplementary Table 1). In both callus and seedling, the peaks of H3K27me3 were mainly enriched in the intergenic regions (Figure 1A). To further explore how H3K27me3 is distributed along the gene region, we plotted the average meta-gene profile from transcription start sites (TSSs) to transcription termination sites (TTSs). We found that in both callus and seedling, H3K7me3 was significantly enriched in the whole gene body, and the deposition of H3K27me3 in seedling was slightly higher than that in callus (Figure 1B). The profile patterns of callus and seedling were relatively consistent, which also indicated the reliability of the ChIP-Seq experimental data and the relative conservation of H3K27me3 (Figure 1B). To investigate the differences in H3K27me3 deposition between callus and seedling tissues and how these differences regulate the expression of tissue-specific genes, we used MACS to determine the preferentially deposited peaks between callus and seedling. Then, 2,714 and 4,562 genes with differential H3K27me3 deposition were identified corresponding to the specific enrichment of H3K27me3 in callus and seedling, respectively (Figure 1C and Supplementary Table 2). To further study the biological processes and molecular functions in which H3K27me3 is involved, GO enrichment analysis was conducted on the genes with differentially deposition of this modification. The results showed that the 4,562 genes with preferential H3K27me3 deposition in the seedling were mainly involved in the processes of regulation of transcription, DNA-templated, response to oxidative stress, and cell wall related (Figure 1D), while the 2,714 genes with higher deposition of H3K27me3 in the callus tended to be involved in defense response, apoptotic process, protein modification (Supplementary Figure 1).

To further analyze the distribution profile of H3K27me3 between callus and seedling genes, we generated a chromatin-based SOM map with clustering regions that contain epigenetic marks to display the differential H3K27me3 deposition in callus and seedling. We mapped the H3K27me3 ChIP-Seq data to the trained SOM map based on the epigenetic signals (Liu et al., 2018) and found that the H3K27me3 signals in the callus were highly consistent with those in the seedling (Supplementary Figure 2). SOM comparison also showed that the units with major differences in H3K27me3 deposition between callus and seedling, and the results showed that H3K27me3 preferentially had higher enrichment scores in seedling than in the callus (Supplementary Figure 1).

### H3K27me3 Shows Decreased Deposition in Callus and Is Capable of Regulating Key Transcription Factors Involved in Meristematic Differentiation

**FIGURE 2:**
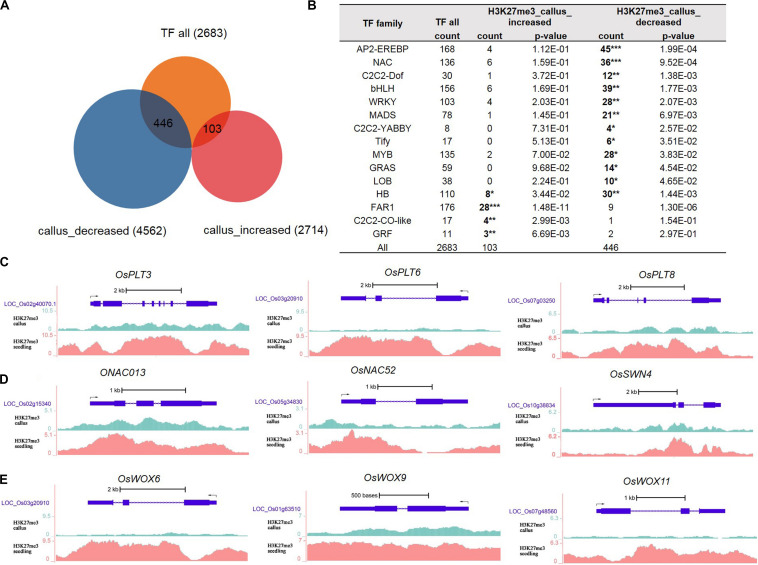
Transcription factor analysis of genes deposited by H3K27me3 in callus and seeding, respectively. **(A)** Venn diagrams for 2,683 TFs and H3K27me3-targeted genes between callus and seedling. **(B)** Transcription factor (TF) families enriched in preferentially deposited H3K27me3 in callus and seedling, respectively. **(C)** Genes in the AP2/EREBP family are associated with H3K27me3 according to data in the UCSC genome browser. **(D)** Genes in the NAC family are associated with H3K27me3 according to the UCSC genome browser. **(E)** Genes in the HB family are associated with H3K27me3 according to the UCSC genome browser.

Gene Ontology analysis results indicated that genes with less deposition of H3K27me3 in the callus were enriched in the process of regulation of transcription. Therefore, we obtained data for 2,683 transcription factors of rice from the plnTFDB database (Riano-Pachon et al., 2007). As shown in Figure 2A, 446 TFs had decreased deposition of H3K27me3 in the callus, while 103 TFs had increased deposition of H3K27me3 in the callus compared with that in the seedling. We performed hypergeometric distribution analysis to examine the enrichment of these genes with differential H3K27me3 deposition among the TF gene families. The results showed that the 446 TFs with decreased deposition of H3K27me3 in the callus mainly belonged to specific gene families such as AP2/EREBP, NAC, C2C2-Dof, bHLH, WRKY MADS, GRAS, and HB (Figure 2B and Supplementary Table 3). In this study, we cite several key transcription factors as examples to suggest that H3K27me3 is deposited at lower levels in the callus, and regulates key transcription factors involved in meristematic differentiation. The UCSC genome browser data showed that three *OsPLT*s belonging to the AP2/EREBP family exhibited lower deposition of H3K27me3 in the callus compared with that in the seedling (Figure 2C). Furthermore, three *OsNAC* genes belonging to the NAC family, namely *OsNAC013*, *OsNAC52*, and *OsSWN4*, exhibited decreased deposition of H3K27me3 in the callus (Figure 2D). In addition, three HB family genes, i.e., *OsWOX6*, *OsWOX9*, and *OsWOX11*, were also shown to exhibit lower deposition of H3K27me3 in the callus (Figure 2E). In rice, *OsWOX6*, *OsWOX9*, and *OsWOX11* are involved in callus formation (Hu et al., 2017).

In addition to the AP2/EREBP, NAC, and HB families, we identified a large number of TF genes with decreased deposition of H3K27me3 in the callus (Supplementary Table 3), which may enable elucidation of callus meristematic differentiation. Considering the MADS family genes as an example, 21 genes showed decreased deposition of H3K27me3 in the callus; among them, *LOC_Os02g36924* (*OsMADS27*) is involved in root system development (Chen et al., 2018), while *LOC_Os02g52340* (*OsMADS22*) is involved in Shoot Apical Meristem (SAM) formation and maintenance (Zhou et al., 2016) (Supplementary Table 3).

### The Deposition of H3K27me3 Inhibits the Transcription Process, and Regulates Oxidative Stress, Cell Wall Modification, and Glucan Synthesis

**FIGURE 3:**
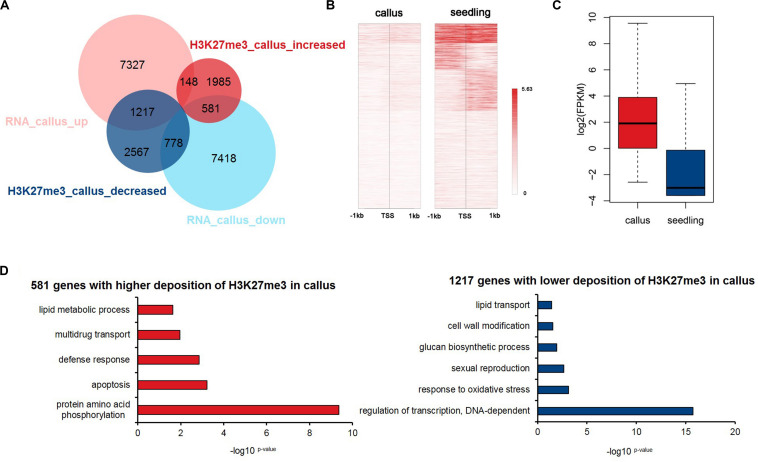
Integrated analysis of H3K27me3-targeted and differentially expressed genes. **(A)** Venn diagrams for H3K27me3-targeted genes and differentially expressed genes between callus and seedling. **(B)** Heatmap of H3K27me3 deposition in 1,217 genes in **(A)** that show lower deposition of H3K27me3 and are highly expressed in the callus. **(C)** The gene expression values are shown for the group of 1,217 genes in **(A)**. **(D)** GO enrichment analysis of 581 and 1,217 genes with higher deposition of H3K27me3 and lower expression levels in callus and seedling, respectively.

Previous studies have shown that epigenetic modifications regulate gene expression. To explore how the deposition of H3K27me3 affects the transcriptome profile, we downloaded RNA-Seq data for the callus and seedling (Zhang et al., 2017b) and obtained 8,692 and 8,777 genes that were highly expressed in the callus and seedling, respectively (Figure 3A and Supplementary Table 4). We compared the groups of H3K27me3-targeted genes and the DEGs, and the results showed that H3K27me3 was negatively correlated with transcription, that is, the genes with higher deposition in the seedling mainly tended to be highly expressed in the callus (1,217 vs. 778), while genes with higher deposition in the callus tended to be highly expressed in the seedling (581 vs. 148) (Figure 3A). To investigate genes that are critical for callus formation and differentiation in more detail, we focused on the 1,217 genes that show lower deposition of H3K27me3 (Figure 3B) but higher expression (Figure 3C) in the callus, indicating a negative relationship between H3K27me3 and gene expression. The main functions of these 1,217 gene were transcriptional regulation, response to oxidative stress, glucan synthesis, cell wall modification (Figure 3D). However, among them, the 581 genes with higher H3K27me3 deposition in the callus were mainly involved in protein phosphorylation, apoptosis, and the defense response (Figure 3D).

### Genome-Wide Profiling of Differential H3K4me3 Deposition Between the Rice Callus and Seedling

**FIGURE 4:**
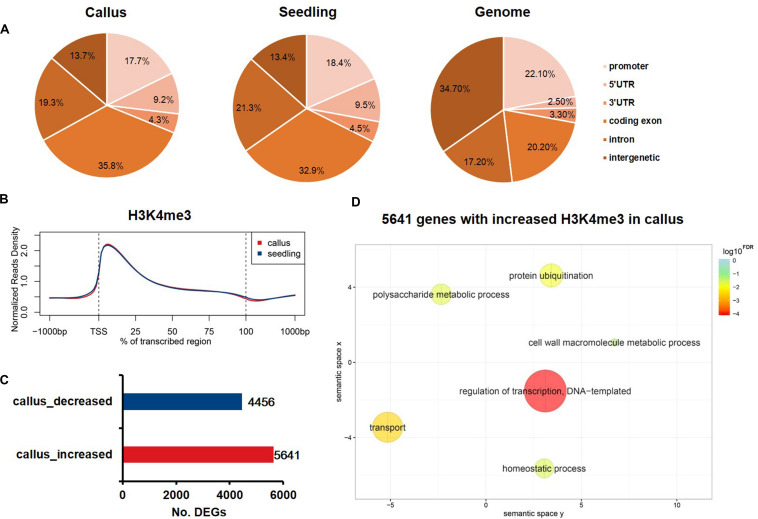
Genome-wide profiling of H3K4me3 in rice callus and seedling. **(A)** Genomic distribution of H3K4me3 peaks within different regions in rice callus and seedling tissues. The rice genome was characterized into six classes that included five genic regions [promoter, 5′ untranslated region (UTR), 3′ UTR, coding exon, and intron] and intergenic regions. **(B)** The distribution of H3K4me3 along all the rice genes in the callus and seedling (from 1-kb upstream regions to 1-kb downstream regions). **(C)** The number of genes with differential deposition of H3K4me3 between callus and seedling. **(D)** GO enrichment analysis of H3K4me3-increased genes in callus by agriGO and REVIGO. The scatterplot shows the cluster representatives in a two-dimensional space derived by applying multidimensional scaling to a matrix of significant GO terms with semantic similarities. Bubble color and size indicate the log10^*FDR*^.

Increasing evidence has shown that changes in H3K27me3 levels are negatively correlated with expression levels, and are critical for the acquisition of pluripotency (Lafos et al., 2011; He et al., 2012). In contrast, H3K4me3 plays an active role in transcription initiation during early embryonic development (Huang et al., 2019). Therefore, we additionally performed ChIP-Seq experiments to study differential H3K4me3 deposition in callus and seedling tissues in order to profile the genome-wide distribution of H3K4me3 in rice. The sequenced reads were mapped onto six genome elements, and, in contrast to H3K27me3, H3K4me3 was found to be mainly enriched in coding exons (Figure 4A). The meta-gene profile showed that H3K4me3 was mainly enriched downstream of TSSs and in the gene body region in both callus and seedling tissues (Figure 2B). We also identified 5,641 genes that had higher H3K4me3 signals, and 4,456 genes that had lower H3K4me3 signals in the callus than in the seedling (Figure 4C). GO enrichment analysis revealed that the 5,641 H3K4me3-increased genes in the callus were associated with regulation of transcription, transport, protein ubiquitination, and cell wall macromolecule metabolic processes, among others (Figure 4D). Further, the 4,456 genes with decreased H3K4me3 deposition were associated with protein phosphorylation, response to oxidative stress, photosynthesis, apoptotic process, etc. (Supplementary Figure 3). The overlap between H3K4me3-targeted genes and DEGs revealed that 2,307 and 2,154 genes with higher deposition of H3K4me3 were highly expressed in callus and seedling, respectively (Supplementary Figure 4A). Therefore, we focused on the 2,307 H3K4me3-enriched and up-regulated genes, which showed increased H3K4me3 signals (Supplementary Figure 4B) and expression levels (Supplementary Figure 4C) in callus. Through GO analysis, we found that these 2,307 genes were associated with regulation of transcription, response to stimulus, etc. (Supplementary Figure 4D), while the 2,154 genes with higher H3K4me3 deposition in the seedling were associated with response to oxidative stress, photosynthesis, apoptosis, etc. (Supplementary Figure 4E).

### Genes Preferentially Expressed in the Callus Were Cooperatively Regulated by H3K27me3, H3K4me3, and DHSs

Previous studies have shown that many genes are activated during callus formation, and that these are subject to dynamic regulation by multiple epigenetic modifications (Lee and Seo, 2018). Although the relationship between each individual modification and the level of activity of various genetic functional elements is understood, their combined effects on gene expression patterns remains to be elucidated. The roles of H3K27me3 in the suppression of gene expression as well as those of H3K4me3 in the activation of gene expression, and DHSs, which are associated with open chromatin structure, in the regulation of callus formation remain to be understood.

**FIGURE 5:**
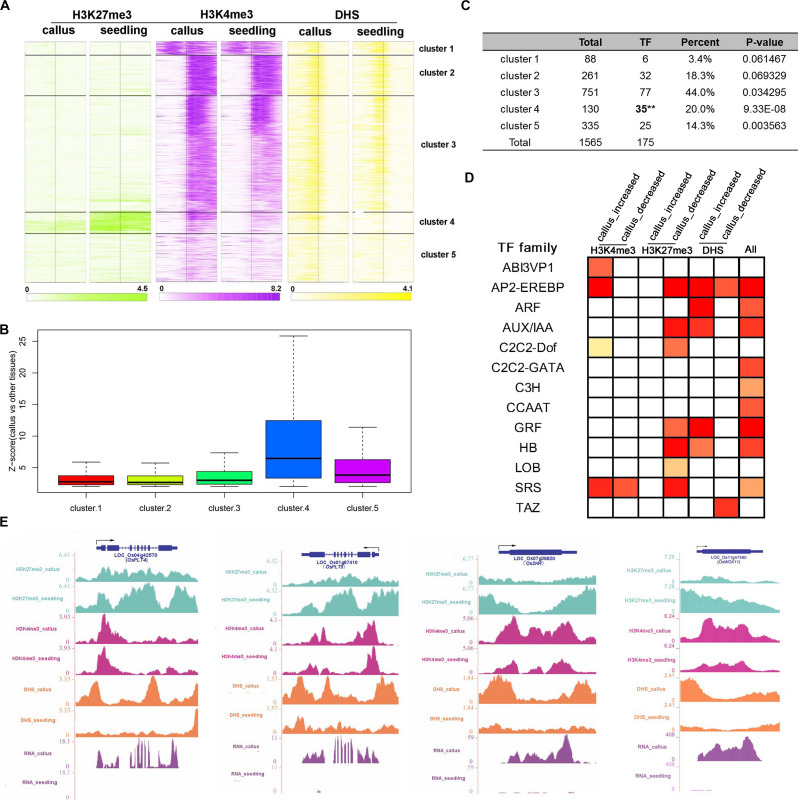
Combination analysis of H3K27me3, H3K4me3, and DHSs near the callus-preferential genes. **(A)** Heatmap of H3K27me3, H3K4me3, and DHSs around the TSSs of 1,587 callus-preferential genes (from 1 kb upstream to 1 kb downstream regions of TSSs). All 1,587 genes were clustered on the basis of their enrichment, using the *k*-means method. **(B)** *Z*-scores (callus vs. other tissues) of 1,587 callus-preferential genes from five clusters in **(A)**. **(C)** TF enrichment analysis of five clusters using the hypergeometric distribution method. **(D)** TF family enrichment analysis of all 175 TFs preferentially expressed in the callus, and TFs with histone mark deposition, using the hypergeometric distribution method. **(E)** UCSC genome browser-based visualization for *OsPLT4*, *OsPLT5*, and *OsSHR*.

In order to investigate the genes that are preferentially expressed in the callus in more detail, we additionally performed transcriptomic analysis of 45 sets of Affymetrix GeneChip arrays in rice; 13 tissues and 17 development stages were analyzed, and finally, 1,565 genes were filtered (Supplementary Table 5). Then, we clustered these genes into 5 clusters according to the characteristics of various epigenetic modifications (Supplementary Table 5 and Figure 5A). In general, these three modifications exhibited a basic feature: H3K27me3 was highly modified in the seedling, and H3K4me3 and DHSs were highly enriched in the callus. This feature was most obvious in cluster 4, which, notably, was also the cluster with the highest degree of transcription factor enrichment (Figures 5A,C). Among the 5 clusters, the highest average expression level of these genes was observed in cluster 4 (Figure 5B), which had lower deposition of H3K27me3 in the callus but higher deposition of H3K27me3 in the seedling (Figure 5A). This also indicated that H3K27me3 played an important role in regulating the function of genes involved in callus development. To further explore the function of these genes, we carried out TF family enrichment analysis of these 175 callus preferentially expressed TFs, and found that these TFs were mainly negatively regulated by H3K27me3 and positively regulated by H3K4me3 and DHSs (Figure 5D). The results obtained by TF enrichment analysis were also consistent with previous results, that is, the genes preferentially expressed in the callus belonged to gene families involved in callus formation or related functions, and included genes encoding the ethylene response binding element protein AP2/EREBP TF family, ARF, AUX-IAA TF families related to auxin, GRF TF family of growth regulators, and HB TF family of homeoboxes (Figure 5D and Supplementary Table 5).

**FIGURE 6:**
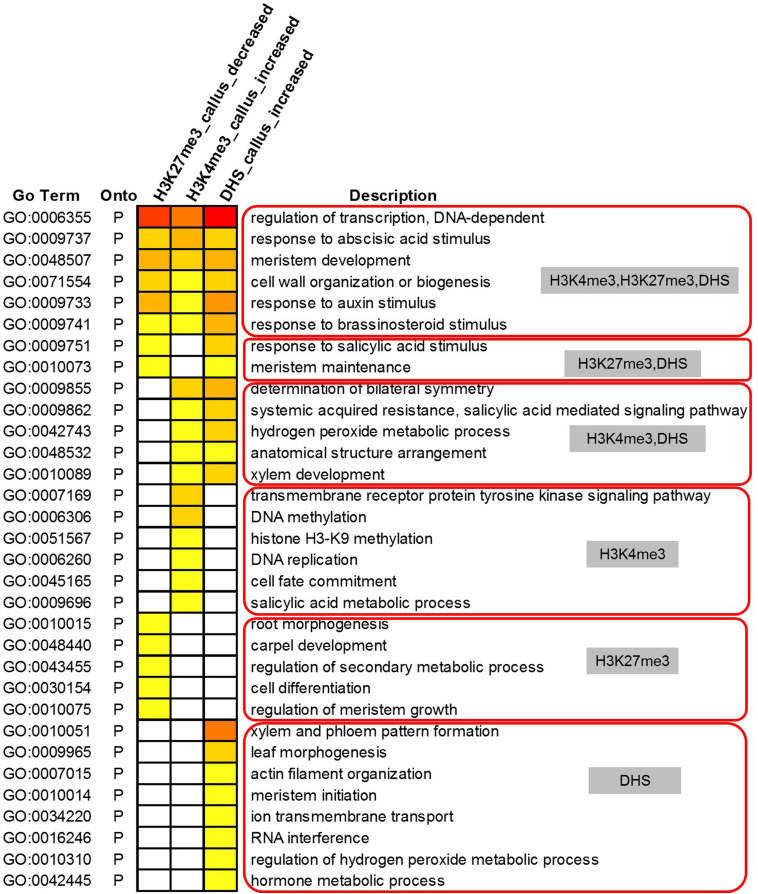
Gene Ontology analysis of 1,565 highly expressed genes with lower deposition of H3K27me3 but higher deposition of H3K4me3 and DHSs in the callus.

We further conducted GO analysis for the 1,565 genes preferentially expressed in the callus that showed lower deposition of H3K27me3 but higher deposition of H3K4me3 and DHSs in the callus (Figure 6). The result showed that H3K27me3, H3K4me3, and DHS regulate the processes of transcription, response to stimulus, cell wall organization, and meristem development. H3K27me3 combined with DHSs regulated the processes of meristem maintenance and response to salicylic acid stimulus. H3K4me3 combined with DHSs regulated the leaf development process and hydrogen peroxide metabolic process. Furthermore, the individual marks regulated specific processes: H3K4me3 was mainly involved in DNA replication, H3K9 methylation, and DNA methylation, while H3K27me3 was mainly involved in organ development and cell differentiation. DHSs were involved in organ development, meristem initiation, ion transmembrane transport, RNA interference, and metabolic processes.

It is known that DNA methylation undergoes substantial changes during callus formation, and which also plays critical roles. To explore the possible relationship of H3K27me3, H3K4me3, and DNA methylation, we downloaded the public data of DNA methylation in callus and seedling (accession number: GSE42410) (Stroud et al., 2013). Here we performed the cluster analysis based on the enrichment of different epigenetic marks. The results showed that the enrichment of H3K4me3 and DNA methylation around the genes was opposite, that is, H3K4me3 was mainly enriched downstream of TSS, while DNA methylation was mainly upstream of TSS, especially in the clusters 2, 5, 6, and 7. And in the cluster 4, there was less deposition of H3K4me3 but higher deposition of H3K27me3 and DNA methylation. In the cluster 8, there was more deposition of DNA methylation around the whole gene body (Supplementary Figure 7). The results implied the synergy or antagonism between DNA methylation and different histone modifications. However, the specific genes regulated by DNA methylation and the specific regulatory mechanism with histone modifications still need further exploration.

### Key Developmental Regulators Were Marked by Bivalent Chromatin, and Tended to Be Active in the Callus but Repressed in Seedling Tissue

**FIGURE 7:**
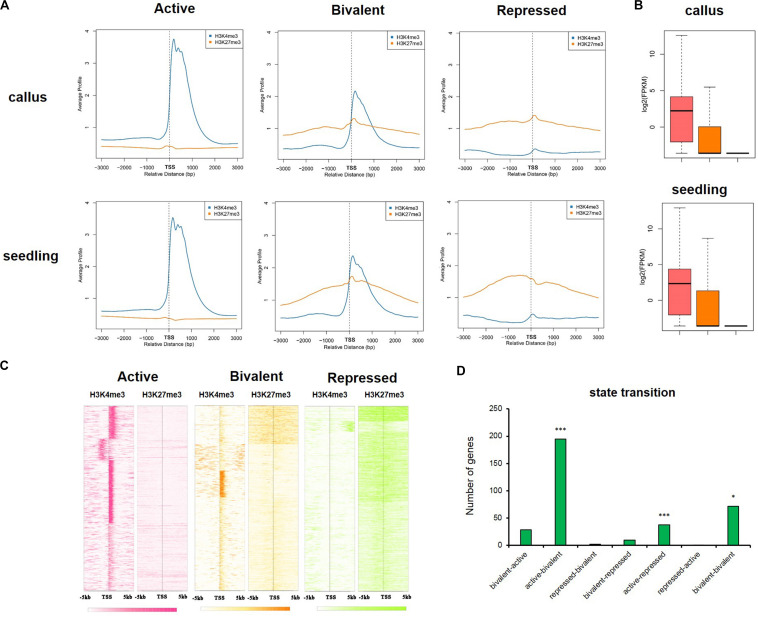
The active, bivalent, and repressed states in callus and seedling. **(A)** The average gene profiles of H3K4me3 and H3K27me3 around TSS (from upstream 3 kb to downstream 3 kb) in the “active,” “bivalent,” and “repressed” states. **(B)** The gene expression profiles of the genes under the three states mentioned in **(A)**. **(C)** The H3K4me3 and H3K27me3 signals around TSS (upstream 5 kb and downstream 5 kb) of the genes in the three aforementioned states in the callus. **(D)** The 7 state transitions of genes preferentially expressed in the callus: “bivalent-active,” genes that are in the bivalent state in the callus, but in the active state in the seedling; “active-bivalent,” genes that are in the active state in the callus, but in the bivalent state in the seedling; “repressed-bivalent,” “bivalent-repressed,” “active-repressed,” “repressed-active,” and “bivalent-bivalent” are the same as defined above. The significance was evaluated by hypergeometric distribution (*P < 0.01, ***P < 0.001).

In animals, there is abundant evidence for the bivalence of developmental genes carrying H3K27me3 and H3K4me3 marks, especially in embryonic stem cells (Bernstein et al., 2006; Vastenhouw and Schier, 2012; Sachs et al., 2013). To investigate the differences in the epigenetic regulatory states of callus and seedling, we defined genes with H3K4me3 only as being in the “active” state, genes with H3K27me3 only as being in the “repressed” state, and genes with both H3K4me3 and H3K27me3 as being in the “bivalent” state (Figure 7A). The average gene profile showed that the active genes had higher deposition of H3K4me3 and lower deposition of H3K27me3 around TSS, and, in the bivalent state, the genes had higher deposition of H3K27me3, but the deposition of H3K4me3 was decreased (Figures 7A,C). We investigated the expression of genes in these different states and found that when only H3K4me3 was present, gene expression was highest, and when only H3K27me3 was deposited, the expression of the gene was significantly inhibited (Figure 7B). Gene expression is regulated by chromatin state; therefore, a transition between chromatin states may be involved in the tissue-specific expression of key developmental genes. We defined the seven different state transitions from callus to seedling (Supplementary Figure 5 and Supplementary Table 6). We previously identified 1,565 genes highly expressed in callus using Affymetrix GeneChip datasets, which were closely related to callus characteristics. We investigated the transition in chromatin state of these 1,565 genes from callus to seedling (Supplementary Table 6) and found that these genes are significantly enriched in three states, i.e., “active-bivalent,” “active-repressed,” and “bivalent-bivalent” (Figure 7D). These three states correspond to expression levels from relative activation to relative inhibition (“active-bivalent,” “active-repressed”) or substantially stable (“bivalent-bivalent”) in the callus, which also indicates that these genes are indeed activated in the callus. For example, *OsLEC1* (*LOC_Os02g49370*) and *OsWOX12B* (*LOC_Os03g20910*), which belonged to the “active-repressed” state (Supplementary Figures 6A,B). *OsLEC1* was reported to be key regulators of meristem identity determination in the development of leaves, panicles and spikelets in both rice vegetative and reproductive development, and *OsWOX12B* was involved in callus formation and it was induced by primarily in the phloem-pole pericycle cells at 2 h after culture (Zhang and Xue, 2013; Hu et al., 2017). In addition, some auxin-response genes, such as small auxin-up RNA gene family member *OsSAUR18* (*LOC_Os04g43740*), and *OsIAA20* (*LOC_Os06g07040*), which was highly expressed in roots before flowering (Rice Full-Length cDNA Consortium et al., 2003) were also in the “active-repressed” state (Supplementary Figures 6C,D). *OsVP1* (*LOC_Os01g68370*) was in the “active-bivalent” state (Supplementary Figure 6E), and it is expressed in rice suspension-cultured cells (Nakagawa et al., 1996) and the Yeast one-hybrid assay and ChIP analyses has proved that OsEMF2b could bind to the promoter of *OsVP1*, therefore affecting H3K27me3 enrichments of *OsVP1* in seedling (Chen et al., 2017). And *WOX11* (*LOC_Os07g48560*) is involved in the process of crown root development which was activated by auxin-YUC-WOX11 module (Zhang et al., 2018) was in the “active-bivalent” state (Supplementary Figure 6F). *OsWOX5* (*LOC_Os03g63510*), which is also involved in callus formation (Hu et al., 2017); *HL6/OsPLT2*, which regulates trichome formation together with *OsWOX3B* (Li and Xue, 2011; Sun et al., 2017); and *OsWOX11*(*LOC_Os11g47580*), which is involved in meristem development through epigenetic reprogramming and it could promote the expression of genes during shoot development through binding to the demethylase JMJ705 (Cheng et al., 2018) belonged to the “bivalent-bivalent” state (Supplementary Figures 6G–I). There were more genes which were in the relative active states in callus (Supplementary Table 6), and they may play important roles in maintaining callus characteristics.

In addition, we also investigated the genes which were in the “repressed-active,” “repressed-bivalent,” and “bivalent-active” states and seldom preferentially expressed in callus, ant these genes were potential regulators of seedling characteristics (Supplementary Table 6).

## Discussion

### The Conservation and Reliability of the ChIP-Seq Datasets Including H3K27me3 and H3K4me3

In this study, we generated data for various tissues and antibodies using the ChIP-Seq experiment to analyze the genes bound by specific antibodies. Comparing the distribution of H3K27me3 and H3K4me3 among different parts of the whole genome in rice, it was found that the distribution of the individual epigenetic modifications in callus and seedling was relatively conserved. A high consistency in distribution in different regions of the whole genome (Figures 1A, 4A) or the deposition of different modifications in the vicinity of the genes (Figures 1B, 4B) was observed. In addition, we used the SOM method for genome-wide mapping of the epigenetic modification H3K27me3 to the already trained SOM map to examine the clustering of H3K27me3 in the whole genome^7^. The results of SOM clustering indicated that H3K27me3 distribution was highly similar in different tissues (Supplementary Figure 2). Although the same modification is highly conserved between different tissues, the genes with differential deposition between callus and seedling were identified by MACS and SUB analysis (Figure 1C and Supplementary Figure 2).

### H3K27me3 Regulates Diverse Pathways Involved in Transcription, Auxin, Oxidative Stress, and Transport

Previous studies have reported that the H3K27me3 modification, which is the major silencing mechanism in plants in *Arabidopsis*, regulates a large number of genes in this plant (4,400) (Zhang et al., 2007). H3K27me3 regulates multiple pathways in callus formation, especially in the process of the transcription regulation. We observed some examples to show that the genes with higher levels of H3K27me3 deposition in the seedling than in the callus may be involved in callus formation (Figures 2C–E). *Plethora* (*PLT*) genes encode transcription factors containing the AP2-domain, with functions in the maintenance of stem cell activity and regulation of the root development of the dicot *Arabidopsis thaliana* (Aida et al., 2004). *OsPLT*s have been reported to regulate the hormone-mediated development of main, crown, and lateral roots of rice (Li and Xue, 2011). *OsPLT3* is mainly expressed in the roots, *OsPLT6* in roots and seeds, and *OsPLT8* in stems (Li and Xue, 2011), with higher deposition of H3K27me3 in the seedling than in the callus (Figure 2C). The ortholog gene *AT1G51190* (*PLT2*) of *OsPLT3* in *Arabidopsis* has been reported to be primarily involved in the establishment of stem cell niche (Aida et al., 2004), the *OsPLT3* was reported to involve in the embryonic development in rice (Khanday et al., 2019). *OsPLT6* is mainly involved in the development of crown roots (Li and Xue, 2011) and the initiation of zygotic development in rice (Rahman et al., 2019), and the ortholog gene *AT5G17430* (*BBM*) in *Arabidopsis* is a master regulator of root and embryo development (Galinha et al., 2007; Horstman et al., 2017). *OsPLT8* is mainly involved in the initiation and development of crown root in rice (Kitomi et al., 2011), and that its ortholog gene *AT4G37750* (*ANT*) in *Arabidopsis* is mainly involved in cell proliferation (Mizukami and Fischer, 2000). *OsNAC013* (Figure 2D) belongs to the NAC family, and its ortholog gene *AT1G56010* (*NAC1*) in *Arabidopsis* is reported to be involved in SAM formation and auxin-mediated lateral root formation (Xie et al., 2000). Another NAC family member, namely *OsNAC52* (Figure 2D), is involved in the response to ABA in abiotic stress (Gao et al., 2010). *OsSWN4* (Figure 2D) also belongs to the NAC family, and is reported to be involved in secondary wall synthesis (Zhong et al., 2011). In rice, *OsWOX6*, *OsWOX9*, and *OsWOX11* (Figure 2E) are involved in the formation of the callus (Hu et al., 2017). The homologous genes *AT5G17810* (*WOX12*) of *OsWOX6*, *AT3G11260* (*WOX5*) of *OsWOX9*, and *AT3G03660* (*WOX11*) of *OsWOX11* in *Arabidopsis* have been reported to be involved in the process of establishing root primordia during *de novo* organogenesis of leaf explants; among them, the expression of *WOX5* is regulated by *WOX11* and *WOX12* (Hu and Xu, 2016). In all, these transcription factors were potential regulators of callus formation, but the specific biological process they were involved in still needs further exploration.

The phytohormone auxin regulates gene expression, and its transport plays a role in many processes during plant growth and development, including embryo and lateral organ formation (Zhao, 2008). Auxin and cytokinin have been widely used in callus production; however, the mechanisms by which they induce callus formation at the molecular level and the apparent regulatory mechanisms of the genes that encode them are poorly understood. Auxin is a well-known inducer of lateral root formation in *Arabidopsis* and some members of the transcription factor family (LBD; also known as ASYMMETRIC LEAVES2-LIKE), including LBD16, LBD17, LBD18, and LBD29, which are downstream of AUXIN RESPONSE FACTOR7 (ARF7) and ARF19 (Okushima et al., 2007). Enrichment analysis of the transcription factor family of genes with callus-preferential expression revealed significant enrichment of the LOB and AUX/IAA transcription factor families (Figure 5D). In our results, it showed that *LOC_Os02g57490*, the ortholog of *LBD16* and *LOC_Os03g14270*, the ortholog of *LBD18* were both have decreased deposition of H3K27me3 in callus compared with seedling, whereas there was no significant difference of the ortholog of *LBD17* and *LBD29* in rice. H3K27me3 profiles of shoot apical meristems and leaves were mapped in *A. thaliana*, and differential gene analysis also showed that genes involved in auxin transport and synthesis showed H3K27me3 deposition in leaves (Lafos et al., 2011).

In addition to the phytohormones auxin, cytokinin, ABA, and ethylene (Su and Zhang, 2014), plant regeneration is also regulated by jasmonate (Zhang et al., 2019; Zhou et al., 2019). Our results also showed that the genes preferentially expressed in the callus were involved in the response to multiple hormone stimuli (Figure 6). In *Arabidopsis*, detached leaves produce jasmonate as a damage signal to activate the expression of *ERF109*, thereby promoting auxin synthesis (Zhang et al., 2019). Jasmonate, the stress hormone, regulates stem cell activation and regeneration through the activation of the stress response protein ERF115 (Zhou et al., 2019), which functions downstream of ERF109. Our ChIP-Seq data showed that the ortholog of *ERF109*, *LOC_Os09g28440*, which was reported to be associated with tillering and panicle branching (Qi et al., 2011) and the ortholog of *ERF115*, *LOC_Os04g32620*, which was involved in response to stress response (Wamaitha et al., 2012; Jin et al., 2018), had higher deposition of H3K27me3 in the seedling than in the callus (Supplementary Table 3). In addition, through GO analysis, we also found that H3K27me3 regulated several genes related to oxidative stress and lipid transport in the seedling (Figures 1D, 3D).

To explore the similarity or differences in molecular regulations of callus between dicot and monocot plants, we downloaded the ChIP data of H3K27me3 in *Arabidopsis*. We compared the differential H3K27me3 deposited genes in *Arabidopsis* to those in rice, and found that the genes with decreased deposition in callus compared to seedling also tended to have decreased deposition in the leaf-to-callus transition. Both the differential genes in rice and *Arabidopsis* were involved in transcription activity, metabolism, multiple hormones. However, there were also some genes in the opposite direction between dicot *Arabidopsis* and monocot rice (Supplementary Table 2). In all, we could infer that the genes and pathways involved in the callus formation were overlapped between dicot and monocot plants, but much differences should be further explored.

### The Deposition of H3K4me3, DHSs, and the Removal of H3K27me3 in Callus Affect Multiple Processes in Callus Formation

Transcription factors (TFs) regulate gene expression by binding to specific *cis*-regulated sequences in the promoters of their target genes (Franco-Zorrilla et al., 2014). In eukaryotes, the binding of transcription factors is highly dependent on the local chromatin structure.

The homeodomain transcription factor WUSCHEL (WUS) is critical for the re-establishment of the bud stem cell niche in *Arabidopsis*. The cytokinin-rich environment initially promotes the removal of the inhibitory histone marker H3K27me3 at the WUS locus in a cell cycle-dependent manner (Zhang T.Q. et al., 2017).

Using transcriptome data combined with epigenetic data, it was found that genes highly expressed in the callus generally exhibited relatively high H3K4me3 deposition, modification of DHSs, and relatively low H3K27me3 modification (Figure 5A); furthermore, the genes regulated by these modifications were mainly transcription factor-encoding genes.

During the establishment of cellular identity, it is necessary to inhibit variable fate to maintain stable cell differentiation and maintenance of identity of specific complex tissues. Consistent with previous studies, inhibitory epigenetic modifications can inhibit important processes in callus to maintain differentiation, and reduced inhibitory modification can promote the progression of cell fate in various directions. The results of this study also indicated that key genes involved in meristems in seedlings, embryogenesis, and dedifferentiation show high H3K27me3 deposition, which was associated with the suppression of meristem status.

In contrast, H3K4me3 plays an active role in the maintenance of the expression of key genes in the callus meristem, such as *OsPLT6*/*LOC_Os11g19060* (Supplementary Table 2) in the embryo and the key gene for cell division (Boutilier et al., 2002). As an important site for recognizing the open state of chromatin, DHSs exhibit TF-binding properties, and the binding of TF to these sites was highly consistent with the region regulated by H3K27me3.

In rice, *LOC_Os11g47580* (*WOX11*) promotes crown root development; in addition WOX11 transcripts are expressed in embryos, seedling SAM, leaf primordia, and young leaves (Zhao et al., 2009, 2015; Cheng et al., 2018). The expression pattern analysis and phenotypic observation indicated that WOX11 stimulates the cell division and growth of meristematic tissues (Cheng et al., 2018). JMJ705 is a demethylase of H3K27me3, and available data have shown that WOX11 can activate gene expression by recruiting JMJ705 to remove the deposition of H3K27me3 near the gene *WOX11* (Cheng et al., 2018). Our results showed that *LOC_Os11g47580* is highly expressed in the callus and has higher H3K4me3 and DHS deposition and lower H3K27me3 deposition (Figure 5E). In *Arabidopsis*, callus formation on CIM medium follows the process of lateral root development (Sugimoto et al., 2010), and the callus can acquire subsequent regenerative capacity by establishing pluripotent root stem cells. Root regulatory factors include PLT1, PLT2, SHR, SCR, and WOX5 (Kareem et al., 2015; Hu and Xu, 2016; Bustillo-Avendano et al., 2018), and lateral root development regulators include LBD16, LBD29, and WOX11 (Okushima et al., 2007; Fan et al., 2012; Xu et al., 2018). We examined the apparent modifications and expression of these ortholog genes in rice using UCSC browser data and found that genes involved in root development were highly expressed in the callus, with lower deposition of H3K27me3 but higher deposition of H3K4me3 and DHSs (Figure 5E).

## Conclusion

In summary, we conducted ChIP-Seq analysis of H3K27me3 and H3K4me3 deposition in callus and seedling tissues; results showed that H3K27me3 was preferentially enriched in seedling, and that multiple processes are regulated through the removal of H3K27me3 deposition in the callus. In particular, H3K27me3 is deposited in various TF families, and this has been reported to be associated with callus characteristics and multiple hormone-based stimuli. In contrast to H3K27me3, higher deposition of H3K4me3 positively regulates callus characteristics. We further performed transcriptomic analysis using 45 sets of Affymetrix GeneChip arrays and finally identified 1,565 genes that were preferentially expressed in the callus. These genes were then associated with multiple epigenetic marks, and it was found that the dedifferentiated callus showed higher deposition of the activation-associated marks H3K4me3 and DHSs, and tended to be in a more active state than seedling. Furthermore, the combination of these three marks may regulate callus characteristics through different pathways. The present study provides novel insights into the putative epigenetic mechanisms involved in callus formation, and provide new resources for the study of cell division and differentiation.

## Data Availability Statement

The H3K4me3 and H3K27me3 datasets generated for this study can be found in PCSD database (http://systemsbiology.cau.edu.cn/chromstates/index.php); the DHSs datasets analyzed for this study can be found in SRA database of NCBI (accession number: PRJNA142219).

## Author Contributions

WX and ZS conceived and designed the project. KZ, CW, HY, and WX performed the experiments. NZ, WX, and ZS analyzed the data. YL, HY, and ZS contributed bioinformatics platform and analysis tools. NZ, KZ, WX, and ZS wrote the manuscript. All authors read and approved the final manuscript.

## Conflict of Interest

The authors declare that the research was conducted in the absence of any commercial or financial relationships that could be construed as a potential conflict of interest.

## Footnotes

1 http://lifescience.roche.com

2 http://www.sigmaaldrich.com

3 http://www.gelifesciences.com

4 http://www.merckmillipore.com

5 http://systemsbiology.cau.edu.cn/chromstates/

6 http://www.qiagen.com

7 http://systemsbiology.cau.edu.cn/chromstates/
