# Supplementary material for: Systematic Analysis of Differential H3K27me3 and H3K4me3 Deposition in Callus and Seedling Reveals the Epigenetic Regulatory Mechanisms Involved in Callus Formation in Rice
Source: Front Genet. 2020 Jul 17;11:766. doi: 10.3389/fgene.2020.00766 (PMC7379484; doi:10.3389/fgene.2020.00766)
Supplement: Supplementary file 1 [file Data_Sheet_1.zip › Data Sheet 1/Data Sheet 1.pdf]

Supplementary Material

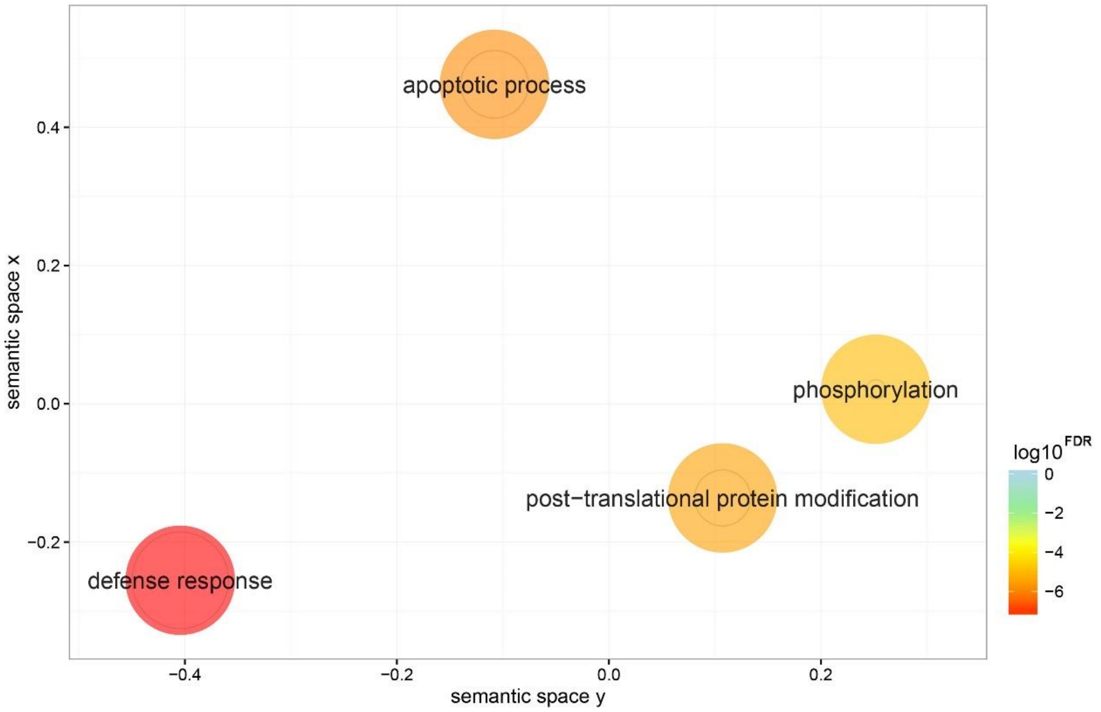

Supplementary Figure 1. Gene Ontology enrichment analysis of 2714 H3K27me3-enriched genes in the callus by agriGO and REVIGO The scatterplot shows the cluster representatives in a two-dimensional space derived by applying multidimensional scaling to a matrix of significant GO terms with semantic similarities. Bubble color and size indicate the log10 ^{FDR}.

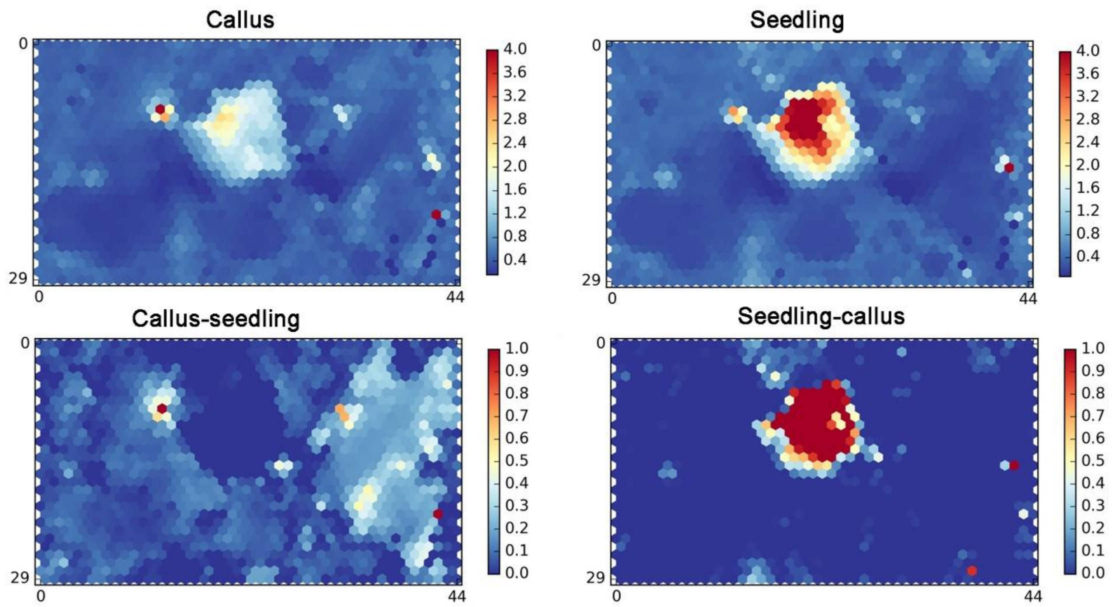

Supplementary Figure 2. **The SOM map analysis for associations between differential deposition of H3K27me3 in callus and seedling** The ChIP-Seq data were mapped to trained Self Organized Map (SOM) map in the PCSD database. "callus-seedling" represents the result obtained by subtracting signals in seedling from signals in callus; "seedling-callus" represents the result obtained by subtracting signals in callus from signals in seedling.

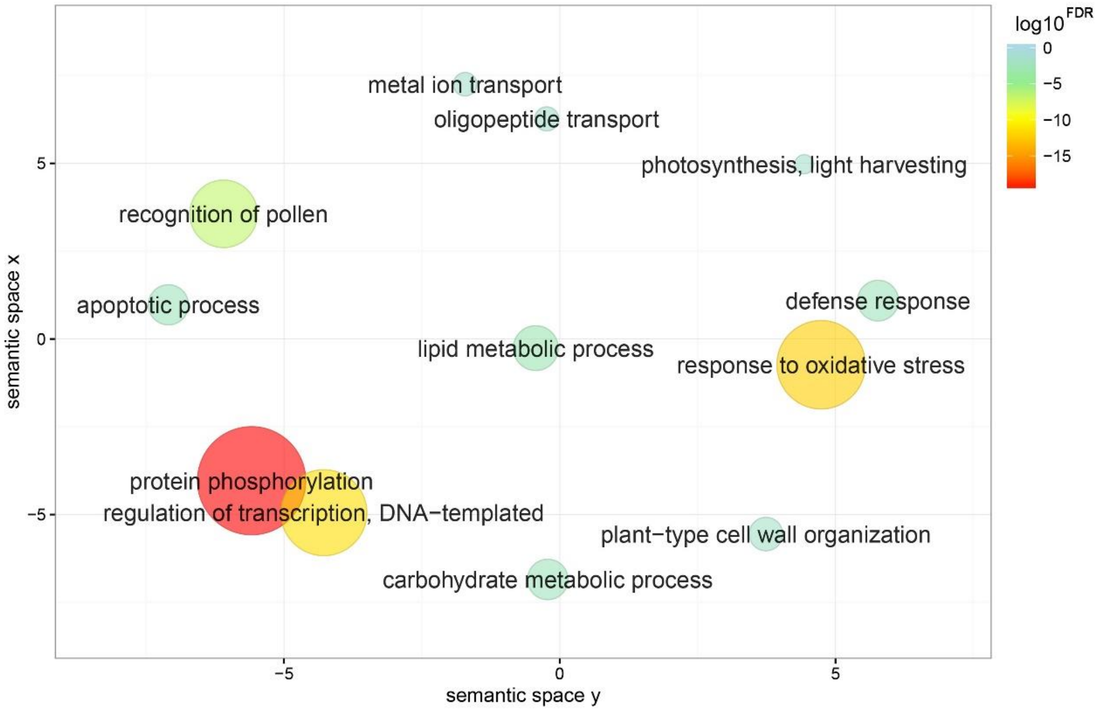

Supplementary Figure 3. Gene Ontology enrichment analysis of 4456 genes with decreased H3K4me3 deposition in callus by agriGO and REVIGO.

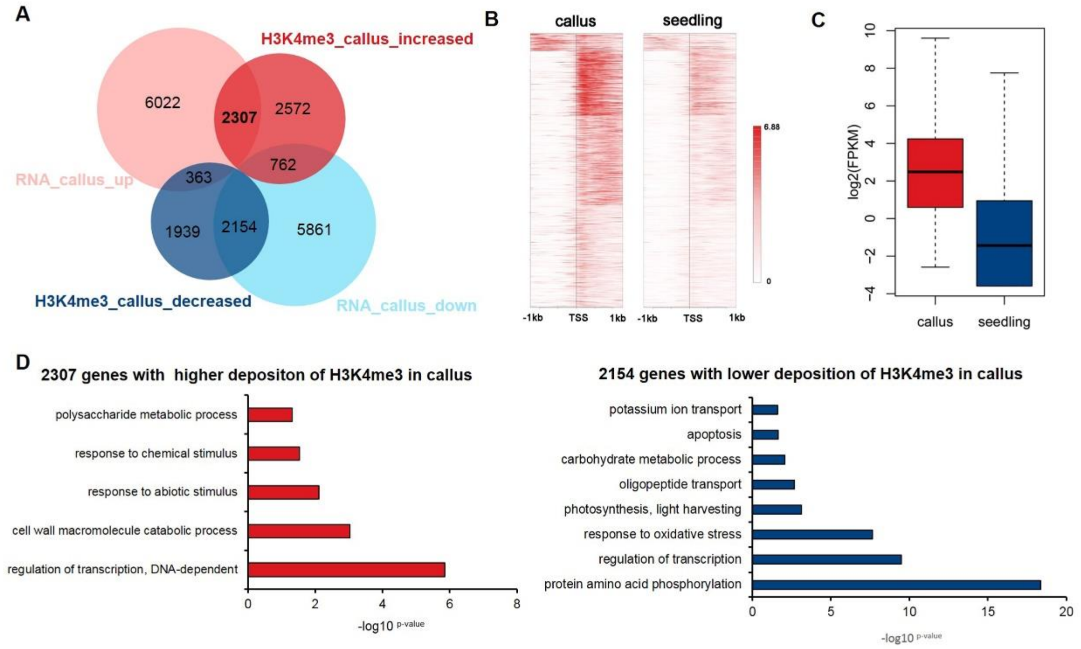

Supplementary Figure 4. The intregrated analysis of H3K4me3-targeted genes and differentially expressed genes

(A) Venn diagrams for H3K27me3-targeted genes and differentially expressed genes between callus and seedling. (**B)** The heatmap of H3K4me3 deposition of 2307 genes in (A) with higher depositon of H3K4me3 that are highly expressed in the callus. (**C)** The gene expression values are shown for the group of 2307 genes in (A). (**D)** GO enrichment analysis of 2307 and 2154 genes with higher deposition of H3K4me3 and lower expression levels in callus and seedling, respectively.

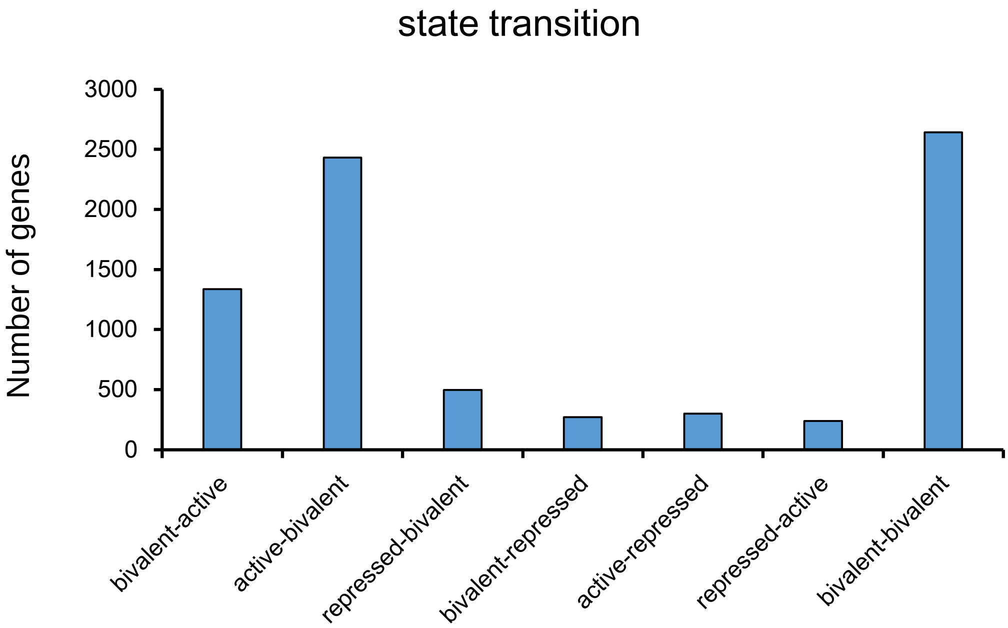

Supplementary Figure 5. The 7 state transitions of all rice genes “Bivalent-k4” indicates genes that are in the bivalent state in the callus, but in the active state in the seedling; “active-bivalent” refers to genes that are in the active state in the callus, but in the bivalent state in the seedling; “repressed-bivalent”, “bivalent-repressed”, “active-repressed”, “repressed-active”, and “bivalent-bivalent” are the same as defined above.

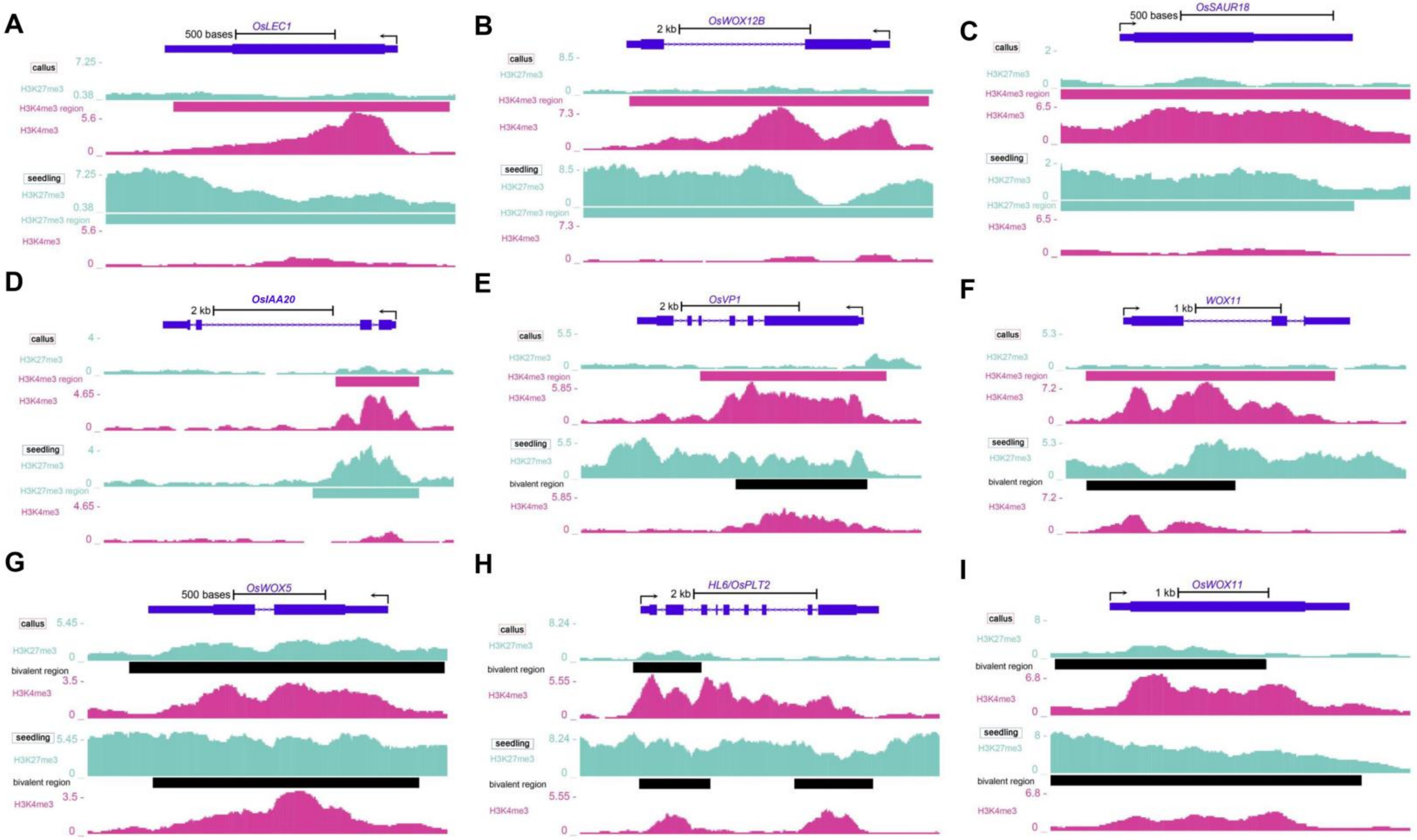

Supplementary Figure 6. UCSC-based visualization of the three states ("active", "bivalent", "repressed") of key developmental genes in callus and seedling.

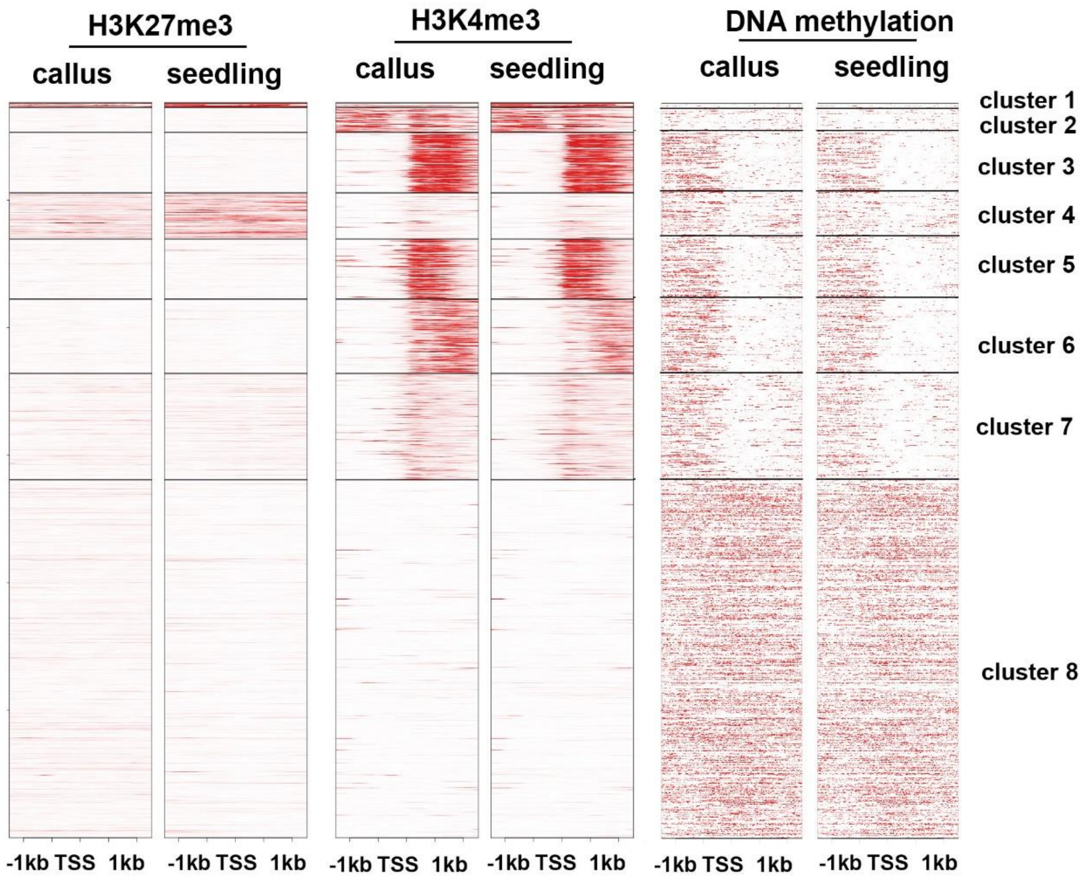

Supplementary Figure 7. The heatmap of H3K27me3, H3K4me3 and DNA methylation.

Supplementary Table 1. Statistical summary of sequencing libraries and the identified peaks number for H3K27me3, H3K4me3 and DHSs in rice callus and seedling.

tissue	summary	H3K4me3	H3K27me3	DHS
callus	Total Reads	22146529	22085450	57037438
	Mapped Reads	21911292	21703120	50531164
	Percentage of total reads	98.94%	98.27%	88.59%
	Number of peaks	33736	15170	35296
seedling	Total Reads	23647717	23813583	42593905
	Mapped Reads	23019603	22983312	40345333
	Percentage of total reads	97.34%	96.51%	94.72%
	Number of peaks	33532	12899	30225

Supplementaly Table 2. Genes with differential deposition of H3K27me3 and H3K4me3 (in separate Excel file).

Supplementary Table 3. The 446 TFs with decreased H3K27me3 deposition in callus compared with that in seedling (in separate Excel file).

Supplementaly Table 4. The RNA-Seq based differentially expressed genes between callus and seedling tissues (in separate Excel file).

Supplementary Table 5. The 1565 genes preferentially expressed in the callus with the deposition of H3K27me3,H3K4me3, and DHSs (in separate Excel file).

Supplementary Table 6. The transitions in chromatin state of rice genes, from callus to seedling (in separate Excel file).
